# The Future of Action Video Games in Psychological Research and Application

**DOI:** 10.3389/fpsyg.2015.01747

**Published:** 2015-11-18

**Authors:** Harun Karimpur, Kai Hamburger

**Affiliations:** Department of Psychology, Experimental Psychology and Cognitive Science, Justus Liebig UniversityGiessen, Germany

**Keywords:** ageing (aging), attention, visual perception, spatial cognition, treatment, dyslexia, action video games, gaming

## Introduction

In recent years, much research has been conducted in order to understand the effects of action video games on mind and behavior. For example, it has been tried to investigate potential links between playing action video games and aggressive behavior (Anderson and Bushman, 2001; Ferguson, 2011), visual selective attention (Green and Bavelier, 2003) or gender differences in spatial cognition (Feng et al., 2007). What we see is that playing certain types of video games in the right doses might enhance several cognitive skills. This can be used in the long run to help those with deficits in these areas, for example the elderly. This article shall emphasize the importance of a holistic and unbiased view in regards to the impact of video games and their possible use. Therefore, it is essential that we desist from antiquated concepts of “typical gamers,” understand the advantages and disadvantages of playing action video games and try to step up efforts in application-oriented research. From our understanding, any physically challenging video game in which reaction time plays a crucial role can be described as an action video game. While there is no generally accepted definition, others define action video games as characterized by the use of violence within these games.

## Action video games, media coverage and public perception

### The role of media

When it comes to the question whether video games (in general) are harmful or not, a heated debate is very likely to start. Especially demographic groups which are not familiar with video games are four to six times more likely to hold a negative opinion regarding that matter (Przybylski, 2014). One reason might simply be the unfamiliarity with gaming in those cohorts. In addition, headlines in the news might also contribute to a negative bias just due to the innuendo effect (Wegner et al., 1981). An innuendo (“games might be harmful”) is a statement about something (“games are harmful”) with a qualifier about the statement (“statement could probably be true”). The innuendo effect occurs when the qualifier has little or no effect. In this case, games would be perceived as harmful. Furthermore, it could be shown that (1) incriminating innuendos had almost the same effect as directly incriminating accusations and (2) the innuendo effect was only minimally reduced, even if the source was one of a bad reputation (Wegner et al., 1981). Further findings show that attempts to reduce this effect (e.g., clarifying statements or major campaigns) might be especially unsuccessful in the case of low processing motivation of the audience (Kim and Chun, 2009).

Earlier this year, news articles depicted a generation of gamers dealing with an early onset of Alzheimer's disease (Call of Duty increases risk of Alzheimer's disease, 2015; Siddique, 2015). Reading this study carefully, one cannot find such causal link. However, it would be wrong to solely blame the media for its coverage. For instance, in terms of health related science it could be shown that not only news are exaggerated but also the press releases (e.g., from the researchers' university) these news are based on (Sumner et al., 2014). The aforementioned news reports, which basically stated that playing action video games leads to Alzheimer's disease, were based on a press release by the Douglas Mental Health University Institute (2015). The lead researcher Dr. Gregory West, inter alia, stated that “gamers rely on the caudate-nucleus to a greater degree than non-gamers.” As a matter of fact, activity of the caudate nucleus was not measured. Notwithstanding the above limitation, journalists might fall for statements like this (fair communication between scientists and administrative staff, politicians, journalists or lay people is currently also addressed as an important future topic in the Cognitive Science community; Gluck and Gray, 2015). Scientists should be aware of their responsibility. Claims unsupported by data and exaggerations of results (e.g., correlations treated as causal relationships) were part of a U.S. Supreme Court ruling in 2011 where the regulation of violent game sales to minors was ruled as unconstitutional (Ferguson, 2013).

### What is a gamer?

Do the average consumers, researchers and journalists have the same concept of a typical gamer? We should be aware that any commonly received pre-millennium concept of a gamer is outdated. According to the Entertainment Software Association (2015), the average video game player is 35 years old and has been playing videos games for 13 years now. About 42% of Americans play video games for 3 h or more per week and around half of them are female, of which about one third plays action video games. German statistics provide similar proportions (e.g., one third playing, 34.5 years on average, 48% female; Bundesverband Interaktive Unterhaltungssoftware, 2014). Thus, the term video game player no longer describes just a few people among the population. Video games are part of our everyday lives, like computers, smartphones or navigation aids. Hence it is crucial that we become clear about the positive and negative outcomes. Today's gamers are tomorrow's elderly, while today's elderly are not yesterday's gamers. This simple but nevertheless important fact shows that research in this area is urgently needed.

## Aging and games

Aging goes hand-in-hand with decline in several psychological areas. In the following, the focus will be exemplary set on three of these areas in regards to aging effects and the possible impact of playing action video games.

### Spatial cognition

Living in an unfamiliar environment such as a retirement home poses a challenge for the elderly. Relatives and caregivers are, for example, often confronted with wandering and getting lost behavior. Even in healthy people the ability to navigate (Morganti et al., 2009) as well as more specifically the ability to acquire spatial knowledge (Jansen et al., 2010) declines with age.

With respect to navigation, especially two different neurophysiological structures play a crucial role, the caudate nucleus and the hippocampus. The former is strongly associated with route learning and response strategies, the latter with wayfinding and spatial strategies (Packard and McGaugh, 1996; Head and Isom, 2010). It is argued that because action video gamers rely more on response strategies than non-action video gamers, playing such type of games might result in an increase of gray matter of the caudate nucleus. Because of the inverse relation between those structures, the authors suggest that a decrease of hippocampal volume might occur, which in turn is associated with Alzheimer's disease (West et al., 2015). Nevertheless, these findings could be explained by reverse causality. Striatal volume could predict the improvement of performance in video games (Erickson et al., 2010). As such, video gamers might feel more attracted to games.

If further studies using quantitative neuroimaging such as volumetry (MRI) could show that there is no negative impact on the hippocampus, certain games could be used or new ones could be designed based on neuroscientific data to enhance route learning skills.

### Perceptual skills and attention

Another set of skills, which declines over time, concerns visual perception. For example, it is known that the useful field of view, which “is defined as the visual area in which information can be acquired within one eye fixation,” declines with age (Ball et al., 1988, p. 1). In one study the useful field of view was measured and compared to the vehicle crash history of the participants. The authors could show that difficulties in the field of visual attention are associated with an increase of vehicle crashes in older drivers (Ball et al., 1993). Another example would be contrast sensitivity. It is long known that elderly observers show a great loss of contrast sensitivity in higher spatial frequencies (Crassini et al., 1988).

Action video games could help to enhance visual perception (Bejjanki et al., 2014) and visual attention, yet the exact mechanisms remain unclear. Focusing on dyslexia, Franceschini et al. (2013) show not only the benefits of action video games but also suggest that the magnocellular-dorsal pathway, impaired in individuals with dyslexia (Gori et al., 2015a), plays an important role as a neural substrate (Franceschini et al., 2013; Gori and Facoetti, 2014, 2015). This hypothesis has successfully been tested by Gori et al. (2015b). In a recent review article, Franceschini et al. (2015) summarize these findings and demonstrate that prevention programs based on action video games could be highly beneficial in regards to developmental dyslexia.

Playing action video games might also enhance the spatial resolution and concomitant the ability to identify objects while distractors are in their immediate vicinity (Green and Bavelier, 2007). Gamers also seem to be better in regards to their attentional capacity (Green and Bavelier, 2003). Additionally, ERP measures show that when put on a visual attention task, action video gamers differ significantly from non-video action gamers in regards to their N2pc component (West et al., 2015), a component which is long known as an indicator of visual attention (Eimer, 1996).

Lastly, video games could also help to treat visual impairments such as amblyopia (“lazy eye”). Making use of the remarkable neuroplasticity during development, eye patching is seen as the standard of treating amblyopia in children. On the contrary, this means that *adult* amblyopia is seen as difficult to treat. However, Li et al. (2011) showed that a treatment consisting of occlusion therapy and game therapy might improve visual acuity in adults. Recent findings also show that a combination of an action video game with perceptual learning and dichoptic viewing improves visual acuity as well as stereopsis (Vedamurthy et al., 2015).

### Task switching

In everyday life, the ability to switch tasks and perform tasks simultaneously is becoming more and more important, especially because of the impact of information technology. Kray and Lindenberger (2000) found by using verbal, figural and numerical material that old and middle-aged adults were less efficient in maintaining and coordinating two different task sets instead of one (it should be mentioned that the so-called switch costs were not as strong for numerical material).

Recent studies show that gaming can be associated with enhancements in regards to task switching abilities. For instance, there seems to be a causal relationship between playing action video games and reduction of switch costs (Green et al., 2012). Neurocognitive plasticity in old age could also be shown in a study with non-action video games (Mayas et al., 2014). Finally, Anguera et al. (2013) demonstrated by using a self-designed three-dimensional racing-game that multitasking not only declines with age but can also be trained with an adaptive version of the game.

## The whole picture

**Figure 1:**
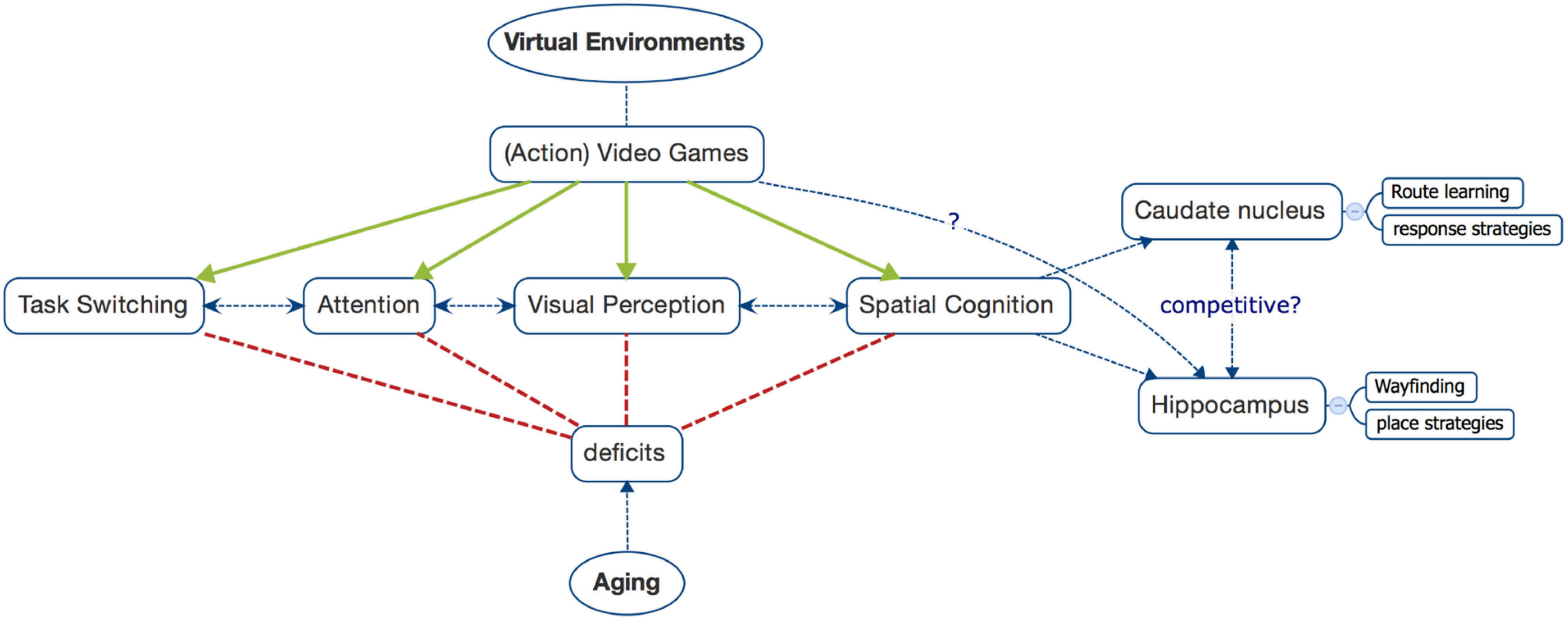
**Visualization of how deficits due to aging might be counteracted by benefits of gaming (please note that special neurological deficits, e.g., due to closed-head injuries or others, are not included here)**. While these links are hypothetical, they are based on the empirical findings presented in this article.

Taken these findings together, the desire to utilize the useful potential of virtual environments emerges (see Figure 1). Especially when looking at demographic change, the actual role of video games within our society and the promising outlook based on preliminary findings, it becomes clear that researchers need to strengthen their efforts in this area. Furthermore, in order to ensure transfer and practical relevance we need to differentiate more precisely within the framework of our research. What kind of game does really help? What are the mechanisms behind these effects? How can these be extracted and implemented in a helpful way in order to develop means of therapeutic use? Finding answers to these questions will not be easy, but we can contribute by also focusing on the positive and stay critically open-minded. Negative effects occur with action video games or multimedia in general, especially when used excessively (“digital dementia”; e.g., Spitzer, 2012). Thus, we also need to find the right dose. Today, many people rely on their smartphones. They definitely can facilitate our work and everyday life, but excessive use might be detrimental as well (e.g., addiction). Scientists and lay people are aware of this, but hardly anybody claims to abandon smartphones, which would be inappropriate. Certainly, video games will not turn into the Fountain of Youth (Cranach the Elder, 1546), nevertheless, they might help generations to come to alleviate some negative effects of aging or neurological impairments in general while ensuring patients' acceptance.

### Conflict of interest statement

The authors declare that the research was conducted in the absence of any commercial or financial relationships that could be construed as a potential conflict of interest.
